# Endoplasmic reticulum stress differentially modulates the IL-6 family of cytokines in murine astrocytes and macrophages

**DOI:** 10.1038/s41598-019-51481-6

**Published:** 2019-10-17

**Authors:** Cristina L. Sanchez, Savannah G. Sims, John D. Nowery, Gordon P. Meares

**Affiliations:** 1 West Virginia University, Department of Microbiology, Immunology, and Cell Biology, Morgantown, WV 26506 USA; 2 West Virginia University, Department of Neuroscience, Morgantown, WV 26506 USA

**Keywords:** Stress signalling, Interleukins

## Abstract

In many diseases, misfolded proteins accumulate within the endoplasmic reticulum (ER), leading to ER stress. In response, the cell initiates the unfolded protein response (UPR) to reestablish homeostasis. Additionally, in response to ER stress, various cell types mount an inflammatory response involving interleukin (IL)-6. While IL-6 has been widely studied, the impact of ER stress on other members of the IL-6 cytokine family, including oncostatin (OSM), IL-11, ciliary neurotrophic factor (CNTF), and leukemia inhibitor factor (LIF) remains to be elucidated. Here, we have examined the expression of the IL-6 family cytokines in response to pharmacologically-induced ER stress in astrocytes and macrophages, which express IL-6 in response to ER stress through different mechanisms. Our findings indicate that, in astrocytes, ER stress regulates mRNA expression of the IL-6 family of cytokines that is, in part, mediated by PKR-like ER kinase (PERK) and Janus kinase (JAK) 1. Additionally, in astrocytes, CNTF expression was suppressed through a PERK-dependent mechanism. Macrophages display a different profile of expression of the IL-6 family that is largely independent of PERK. However, IL-6 expression in macrophages was dependent on JAK signaling. Overall, this study demonstrates the cell-specific and differential mechanisms controlling expression of the IL-6 family of cytokines in response to ER stress.

## Introduction

The interleukin (IL)-6 family of cytokines has pleiotropic effects on immune function, metabolism, development, tissue repair, and neural function. This family of cytokines include IL-6, IL-11, oncostatin M (OSM), leukemia inhibitory factor (LIF), cardiotrophin (CT-1), and cardiotrophin-like cytokine (CLC) that bind to their respective receptors. Common among these proteins is the use of the co-receptor glycoprotein (gp)130 either as a homodimer interacting with IL-6R or IL-11R or as a heterodimer with other IL-6 receptor family members to initiate intracellular signaling through the Janus kinase (JAK) / signal transducers and activators of transcription (STAT) pathway upon receptor ligation^1^. Cytokine-dependent activation of JAKs (JAK1, JAK2, JAK3 and Tyk2), which are tyrosine kinases, leads to phosphorylation-dependent activation of STATs (STAT1, 2, 3, 4, 5a, 5b and 6). STATs are latent cytoplasmic transcription factors that, upon phosphorylation, homo- or heterodimerize, translocate to the nucleus, and drive gene expression^2,3^. The IL-6 family signals primarily via activation of STAT3; however, other STATs can be activated at lower levels^1^. STAT activation regulates a wide variety of genes that modulate cellular functions such as proliferation, differentiation, and effector functions to shape an immune response.

Given the similarities in intracellular signaling, it is not surprising that the IL-6 cytokines have overlapping functions. IL-6, IL-11, OSM, LIF, and CNTF all have the ability to induce an acute phase response^4^. Despite similarities in signaling, the IL-6 family members can also elicit different or even opposite effects. For example, IL-6 worsens the multiple sclerosis animal model of experimental autoimmune encephalomyelitis (EAE) while CNTF, LIF, and IL-11 are protective^4–7^. Additionally, aberrant IL-6 and JAK/STAT signaling is implicated in several disease states including autoimmunity and cancer^8–10^.

The endoplasmic reticulum (ER) is a critical signaling and organizing hub for immune function through the regulation of protein folding and secretion, Ca^2+^ levels, and mitochondrial – ER contacts^11,12^. If the protein folding capacity of the ER is disrupted or overwhelmed, excessive misfolded proteins can accumulate within the ER lumen leading to ER stress. In response, the cell will initiate the unfolded protein response (UPR) aimed at restoring homeostasis^13^. The UPR signaling cascade is initiated by three sensor molecules; inositol requiring enzyme (IRE) 1, protein kinase R (PKR)-like endoplasmic reticulum kinase (PERK), and activating transcription factor (ATF) 6. The UPR induces activation of these molecules to initiate downstream signaling aimed at restoring homeostasis or initiating apoptosis if the stress is not resolvable^14^. PERK is a trans-ER membrane serine/threonine kinase that phosphorylates eukaryotic initiation factor 2α (eIF2α), which leads to selective suppression of protein translation. This is an adaptive response to reduce new polypeptides entering the ER to be folded^15^. There is a subset of proteins including transcription factors and chaperones, which are not suppressed, but rather increased by P-eIF2α-induced translational suppression. For example, ATF4 and C/EBP homologous protein (CHOP) are selectively upregulated in response to UPR activation, and continuous translation of these molecules is proapoptotic^16^. Recently, a small molecule, integrated stress response inhibitor (ISRIB), was identified that reverses the ER stress-induced translational block by enhancing eIF2B activity^17^.

Chronic UPR activation has been reported in many diseases, including diabetes, cancer, and neurodegeneration^18–20^. Similarly, inflammation is a hallmark of chronic diseases. ER stress can directly promote inflammatory gene expression, including proinflammatory cytokines such as IL-6^21^. In macrophages, ER stress-induced IRE1 activation drives a pathway involving nucleotide-binding oligomerization domain-containing protein (NOD1/2) and the transcription factor X-box binding protein 1 (XBP1) leading to IL-6 expression^22,23^. Astrocytes are the most abundant glial cell in the CNS and key regulators of neuroinflammation^24^. ER stress-induced IL-6 production in astrocytes differs from macrophages in that it requires PERK and JAK1, but is independent of IRE1 and nuclear factor-κB (NF-κB)^25,26^. Additionally, IL-6 expression in astrocytes does not rely on ATF4^26^, which is distinct from endothelial cells that use both ATF4 and XBP1^27^. While ER stress-induced IL-6 expression has been widely studied, far less is known about other members of the IL-6 family. Previous work has shown that ER stress in intestinal epithelial cells can promote production of IL-6 and IL-11 that can drive STAT3 phosphorylation in an autocrine-dependent fashion^28^. Here, we have examined the regulation of the IL-6 family of cytokines in primary astrocytes and macrophages in response to classic pharmacological inducers for ER stress.

## Results and Discussion

**Figure 1:**
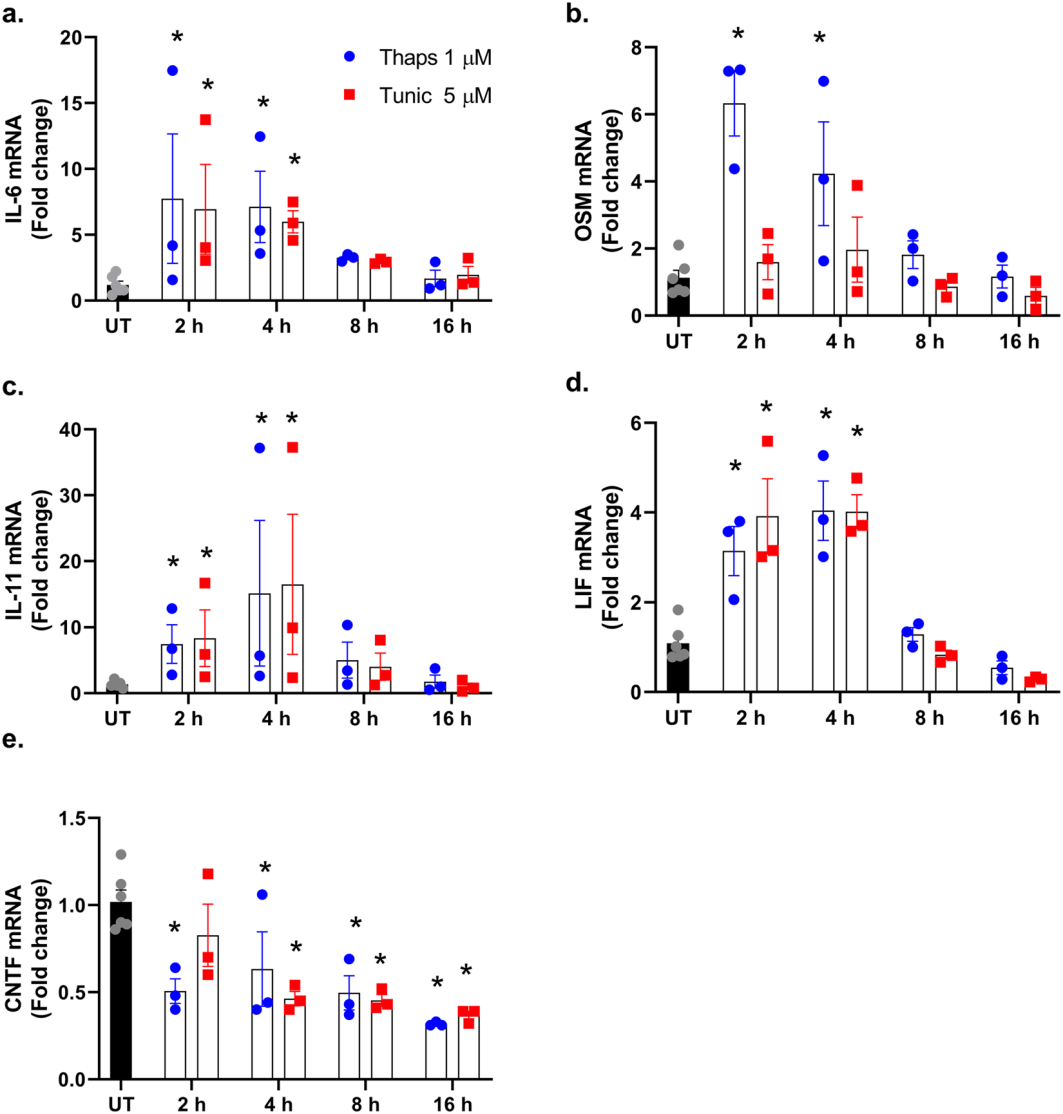
Time-course of the IL-6 cytokine family expression in astrocytes during ER stress. (**a**–**e**) Wild type primary murine astrocytes were stimulated with 1 µM thapsigargin (thaps) or 5 µM tunicamycin (tunic), and mRNA expression was analyzed by qPCR at the indicated time points. Untreated (UT). N = 3, *P ≤ 0.05.

To test how ER stress effects the expression of the IL-6 family of cytokines, primary murine astrocytes were stimulated with thapsigargin (thaps) or tunicamycin (tunic) for 2–16 h. As shown in Fig. 1a–d, ER stress transiently and significantly increased IL-6, OSM, IL-11, and LIF. In contrast, thaps and tunic significantly suppressed CNTF expression (Fig. 1e).

**Figure 2:**
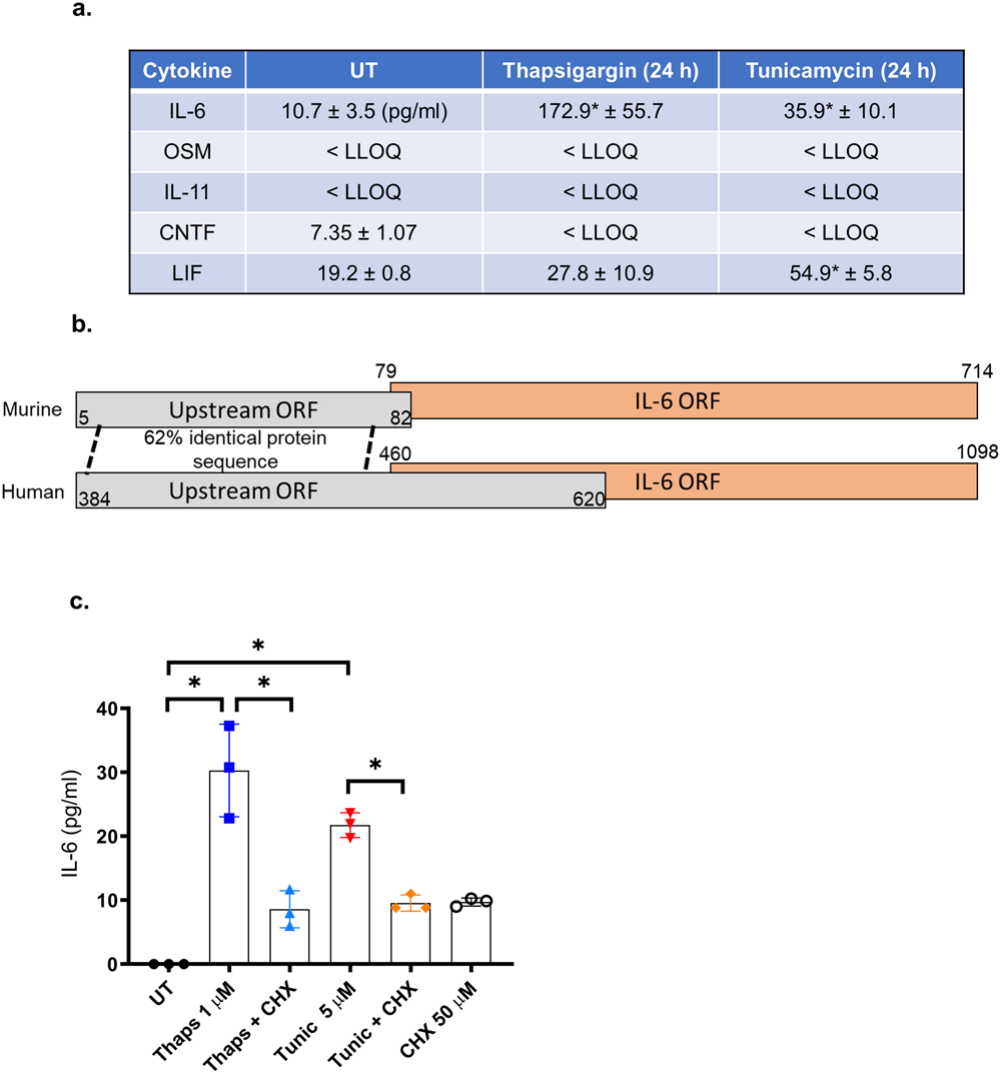
Regulation of IL-6 family proteins during ER stress. (**a**) Wild type primary murine astrocytes were stimulated with 1 µM thaps or 5 µM tunic for 24 h. The cell culture media were then analyzed by ELISA. Lower limit of quantitation (LLOQ). (**b**) Illustration of the murine and human IL-6 transcript showing the presence of a predicted upstream open reading frame (ORF). (**c**) Primary astrocytes were treated with cycloheximide (CHX) (50 µM) for 30 min followed by the addition of thaps (1 µM) or tunic (5 µM) for 24 h. Media were then analyzed by ELISA for IL-6. Data are means ± SD, N = 3, *P ≤ 0.05.

ER stress suppresses protein translation through PERK-dependent phosphorylation of eIF2α^15^. Therefore, we next tested if ER stress could stimulate changes in the IL-6 family of cytokines at the protein level. Primary astrocytes were stimulated with thaps or tunic for 24 h and protein levels were measured by ELISA. Consistent with previous reports, IL-6 protein was strongly increased. LIF was also modestly increased in response to tunic. However, OSM and IL-11 were not increased. CNTF levels were reduced, consistent with mRNA data (Fig. 2a). These data indicate that IL-6 is not suppressed by translational repression while the expression of OSM, IL-11 and CNTF is prevented. Upregulation of OSM and IL-11 mRNA likely reflects an anticipatory response so that the cell is poised to produce these neuroprotective cytokines^29,30^ upon resumption of protein translation. One mechanism known to allow translation during eIF2α phosphorylation is the presence of an upstream open reading frame (ORF)^16^. eIF2α phosphorylation reduces the ribosomal acquisition of initiator methional-tRNA leading to readthrough of the upstream ORF and increased probability of initiating translation at a downstream coding region^16^. Using *in silico* analysis, we have identified that both human and murine IL-6 contain a predicted upstream ORF that overlaps the translational start site and part of the IL-6 coding region (Fig. 2b). This organization is similar to CHOP and ATF4 which are translationally regulated by upstream ORFs^31,32^. OSM, LIF and IL-11 do not contain an upstream ORF, while CNTF contains a non-overlapping upstream ORF. These data indicate that IL-6 can be translated under both translationally competent and repressed states. To confirm that IL-6 is translated and not released from preformed stores, we inhibited translation with cycloheximide (CHX). As shown in Fig. 2c, CHX attenuates IL-6 protein production in response to ER stress. These data indicate that IL-6 can be translated under conditions in which eIF2α is phosphorylated. These data are consistent with previous work showing that herpes simplex virus drives protein kinase R-dependent and CHX sensitive IL-6 expression^33^. Importantly, PKR also phosphorylates eIF2α during viral infection^34^, further supporting that IL-6 translation is resistant to P-eIF2α-mediated repression. We also noted that the upstream ORF shared substantial homology at the amino acid level between murine and human sequences. This may indicate a functional role for the product of these ORFs.

**Figure 3:**
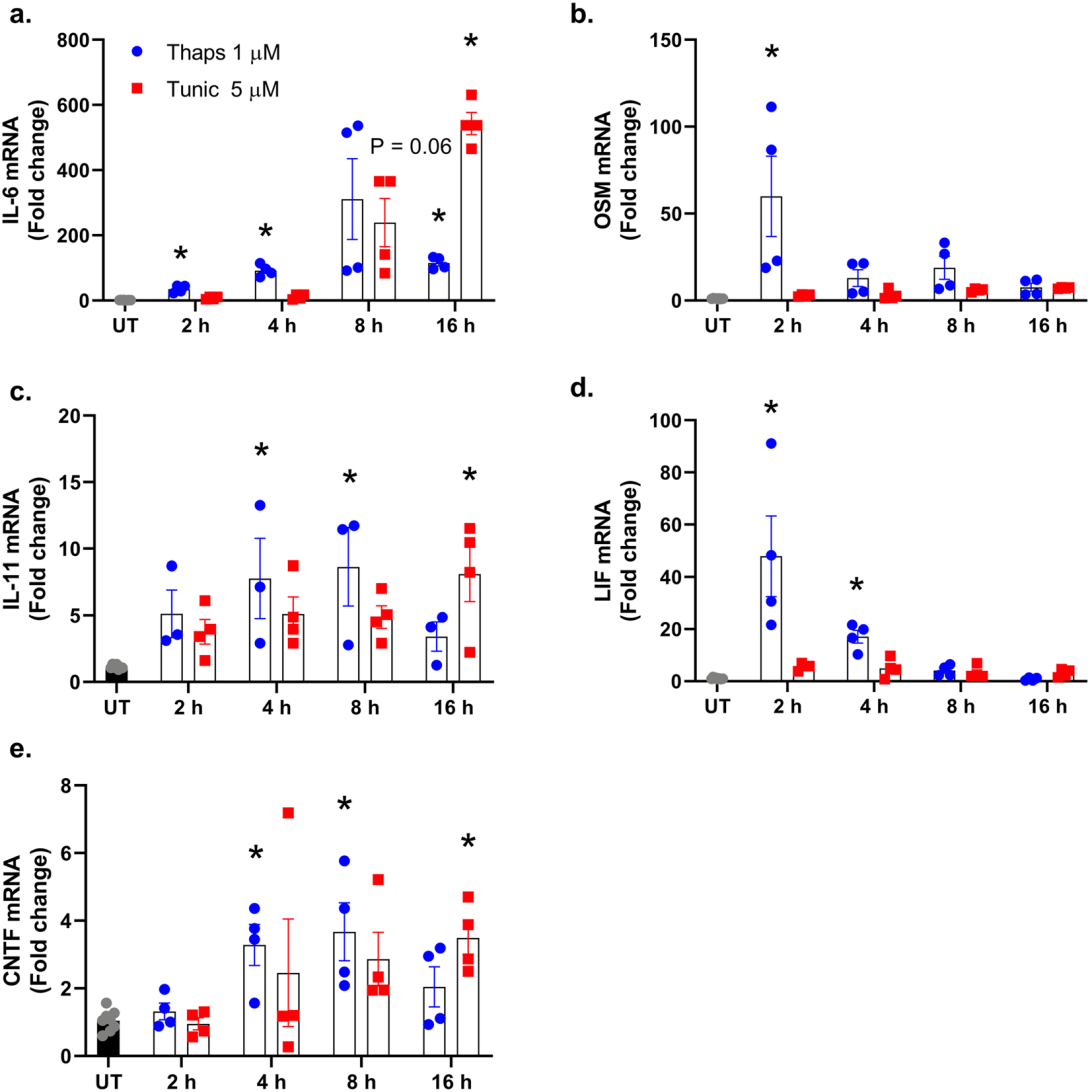
Time-course of the IL-6 cytokine family expression in bone marrow-derived (BMD) macrophages during ER stress. (**a**–**e**) Wild type BMD macrophages were stimulated with 1 µM thapsigargin (thaps) or 5 µM tunicamycin (tunic), and mRNA expression was analyzed by qPCR. N = 4, *P ≤ 0.05.

Next, we examined the impact of ER stress on the expression of the IL-6 family of cytokines in primary macrophages. As shown in Fig. 3a, thaps and tunic increased IL-6 expression as expected. IL-11 was also increased by both thaps and tunic, while OSM and LIF were increased only by thaps (Fig. 3b–d). In contrast to astrocytes, CNTF was significantly increased in macrophages (Fig. 3e). These data indicate that the expression of the IL-6 family of cytokines is differentially regulated depending on the stimulus and cell type.

**Figure 4:**
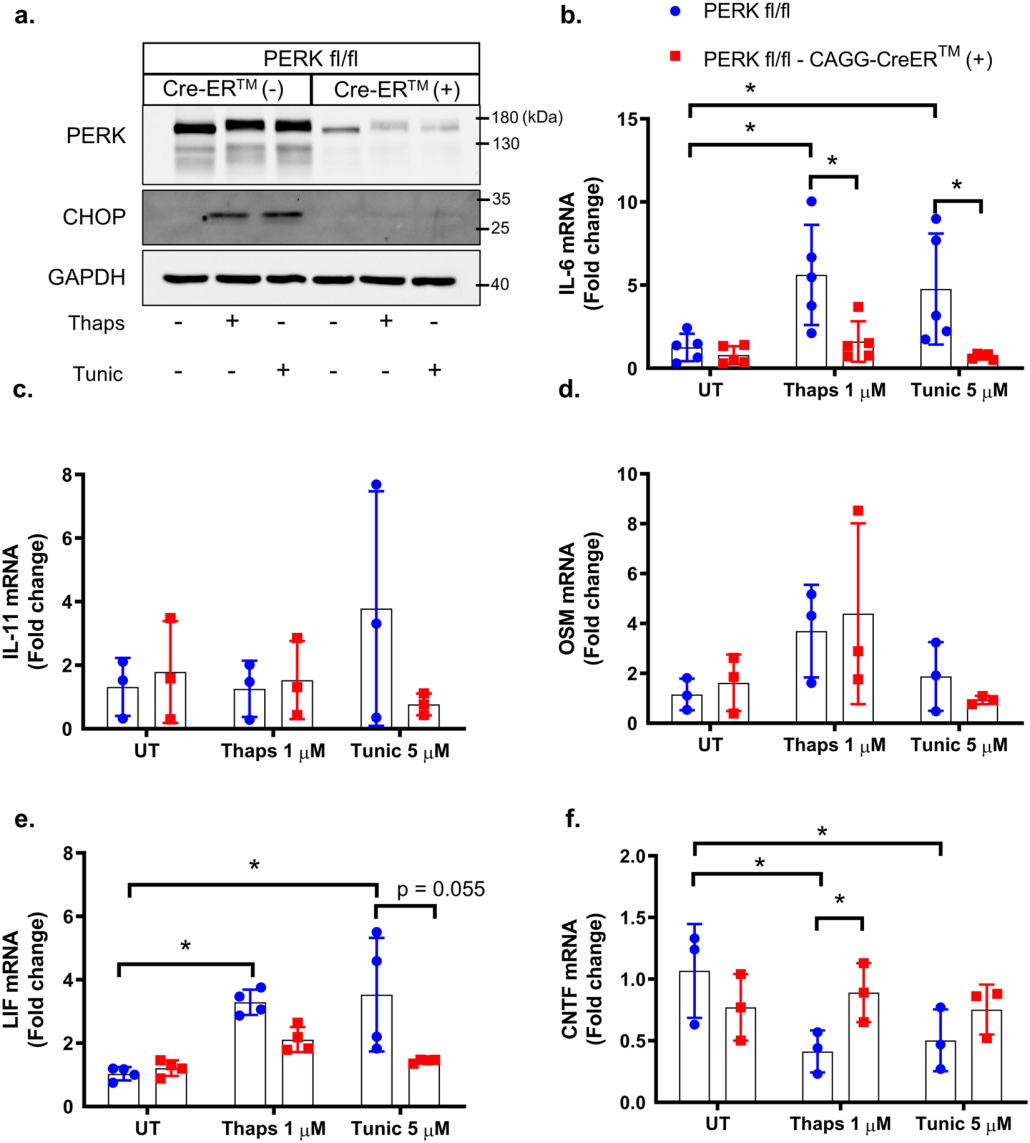
PERK-dependent regulation of the IL-6 family in astrocytes. (**a**) Astrocytes were isolated from PERK^fl/fl^ and PERK^fl/fl^ × CAGG-CreER^TM^ littermates. Primary murine astrocytes were pre-treated with 1 μM 4-OH-tamoxifen for 48 hours to delete PERK. Subsequently, cells were treated with 1 μM thaps and 5 μM tunic for 4 hours followed by immunoblotting to confirm PERK deletion. (**b**–**f**) Cells were isolated and treated as in (**a**), and mRNA expression was analyzed by qPCR. N = 3–5, *P ≤ 0.05. Full-length blots are presented in Supplementary Fig. 1.

**Figure 5:**
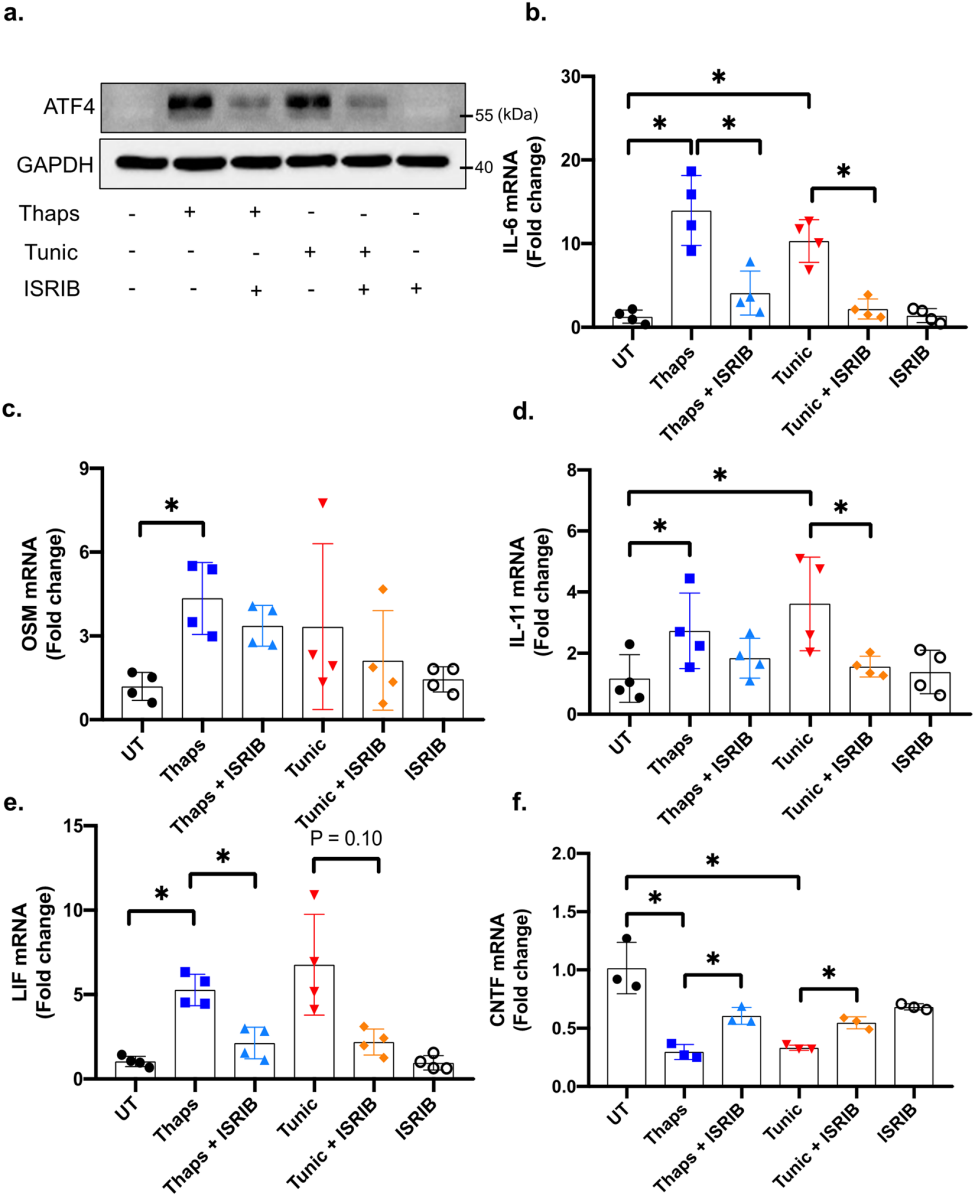
Translational repression regulates the IL-6 cytokine family in astrocytes. (**a**) Primary murine astrocytes were pre-treated with 0.5 μM ISRIB for 30 minutes prior to ER stress stimulation with 1 μM thaps or 5 μM tunic for 4 hours followed by immunoblotting. (**b**–**f**) Cells were treated as in (**a)**, and mRNA expression was analyzed by qPCR. N = 3–4, *P ≤ 0.05. Full-length blots are presented in Supplementary Fig. 2.

Our previous work has shown that ER stress-induced IL-6 expression is PERK-dependent in astrocytes^25^. To test if PERK also regulates other members of the IL-6 family, PERK was deleted from PERK-floxed astrocytes using tamoxifen-inducible cre. As shown in Fig. 4a, PERK was effectively deleted leading to attenuation of CHOP expression. The electrophoretic mobility shift of PERK in response to ER stress (Fig. 4a) is consistent with hyperphosphorylation and activation^35^. PERK deletion abrogated ER stress-induced IL-6 expression and reduced LIF expression, but had no effect on IL-11 or OSM expression (Fig. 4b–e). PERK deletion also rescued ER stress-induced suppression of CNTF expression (Fig. 4f). These data show that PERK drives IL-6 expression while also suppressing CNTF. As such, targeting PERK has the potential to reduce the pro-inflammatory effects of IL-6 while enhancing CNTF, which has neurotrophic and neuroprotective properties^36^. Based on our findings that PERK regulates the IL-6 family in astrocytes, we anticipated that translational repression may similarly regulate gene expression. To test this, we treated astrocytes with the small molecule ISRIB, which attenuates the P-eIF2α-induced translational block^37^. As shown in Fig. 5a, ISRIB effectively suppresses ER stress-induced ATF4 expression, consistent with previous findings^37^. ISRIB potently suppressed ER stress induced IL-6 expression and attenuated IL-11 and LIF expression, but had no effect on OSM induction (Fig. 5b–e). Consistent with the effects of PERK deletion, ISRIB also partially rescued CNTF expression (Fig. 5f).

**Figure 6:**
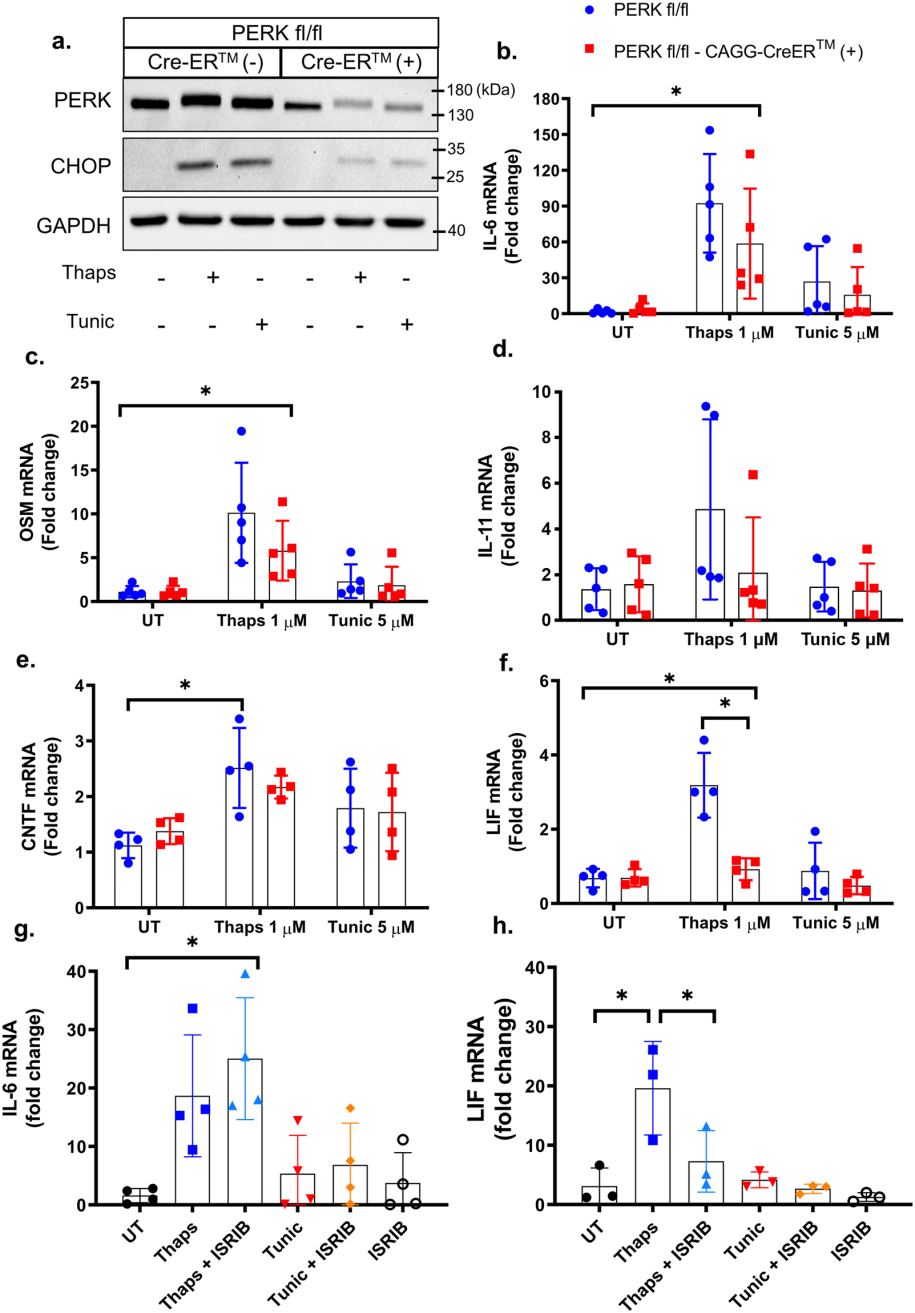
PERK-dependent regulation of the IL-6 family in macrophages. Bone marrow-derived macrophages were isolated from PERK^fl/fl^ and PERK^fl/fl^ × CAGG-CreER^TM^ littermates. (**a**) Macrophages were pre-treated with 1 μM 4-OH-tamoxifen for 48 hours to delete PERK. Subsequently, cells were treated with 1 μM thaps and 5 μM tunic for 4 hours followed by immunoblotting. (**b–f**) Cells were treated as in (**a**) and mRNA expression was analyzed by qPCR. (**g**,**h**) Wild-type macrophages were pre-treated with 0.5 μM ISRIB for 30 minutes prior to ER stress stimulation with 1 μM thaps or 5 μM tunic for 4 hours followed by qPCR. N = 3–5, *P ≤ 0.05. Full-length blots are presented in Supplementary Fig. 3.

To test if PERK has a similar role in macrophages, we again deleted PERK using tamoxifen-inducible cre. PERK and CHOP expression were both reduced in the knockout macrophages (Fig. 6a). In contrast to astrocytes, PERK deletion had a minimal and non-significant effect on ER stress-induced expression of IL-6, OSM, IL-11, and CNTF (Fig. 6b–e). We noted that IL-11 was highly variable in macrophages from PERK transgenic animals (Fig. 6d), making conclusive evaluation difficult. This may be due to the genetic background of the mice or from the pretreatment with tamoxifen. PERK deletion did significantly suppress expression of LIF in macrophages (Fig. 6f). These data indicate that astrocytes and macrophages utilize different mechanisms to control expression of the IL-6 family of cytokines. These data are consistent with previous work showing that thaps-induced IL-6 expression is IRE1-dependent in macrophages and is unaffected by PERK inhibition^22,23^. The molecular basis for this cell type-dependent IL-6 regulation is currently unknown. One possibility is that differential epigenetic modifications allow either PERK- or IRE1-activated transcription factors to preferentially access the IL-6 promoter to drive gene expression. Previous work has shown that, in neutrophils, the IL-6 locus undergoes stimulus-induced chromosomal remodeling to promote IL-6 transcription. Additionally, the chromatin organization of the IL-6 locus was found to differ between neutrophils and monocytes^38^, showing that cell-specific genome structure influences IL-6 expression. A similar effect may occur in astrocytes and macrophages resulting in the utilization of different transcription factors. We next tested if ISRIB has similar effects as PERK deletion in macrophages. As expected, ISRIB had no effect on ER stress-induced IL-6 expression (Fig. 6g). However, ISRIB significantly suppressed LIF expression, consistent with PERK deletion (Fig. 6h). Together, these data suggest that PERK activation and translational suppression influences LIF but not IL-6 expression in macrophages.

**Figure 7:**
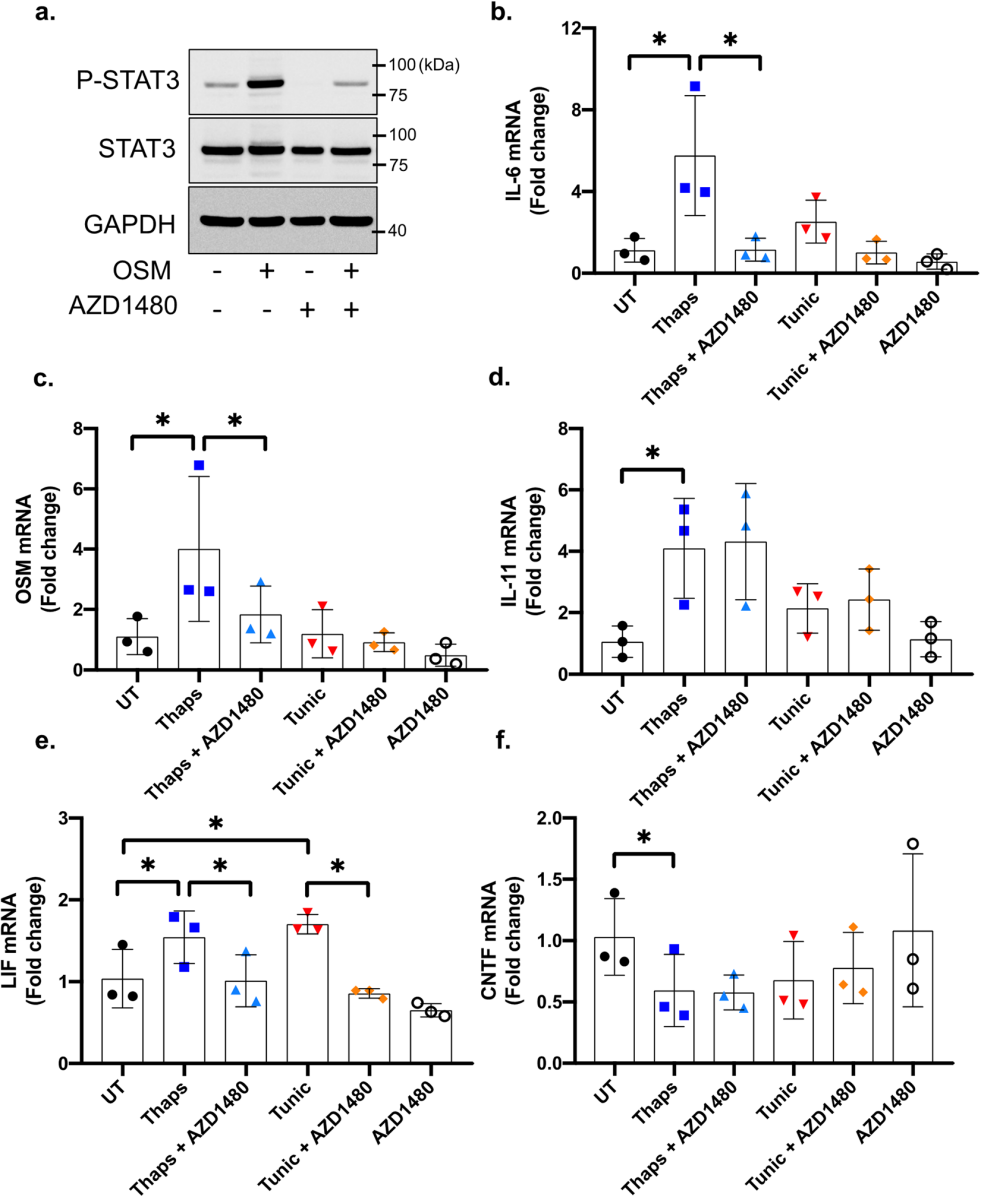
JAK-dependent regulation of the IL-6 cytokine family in murine astrocytes. (**a**) Primary murine astrocytes were pre-treated for 30 min with the JAK1/2 inhibitor, AZD1480 (2 µM) followed by stimulation with 2.5 ng/ml of OSM. Cell lysates were then immunoblotted. (**a**–**f**) Cells were pretreated with AZD1480 for 30 min before stimulation with 1 μM thaps and 5 μM tunic for 4 hours. mRNA expression was analyzed by qPCR. *p ≤ 0.05, N = 3. Full-length blots are presented in Supplementary Fig. 4.

**Figure 8:**
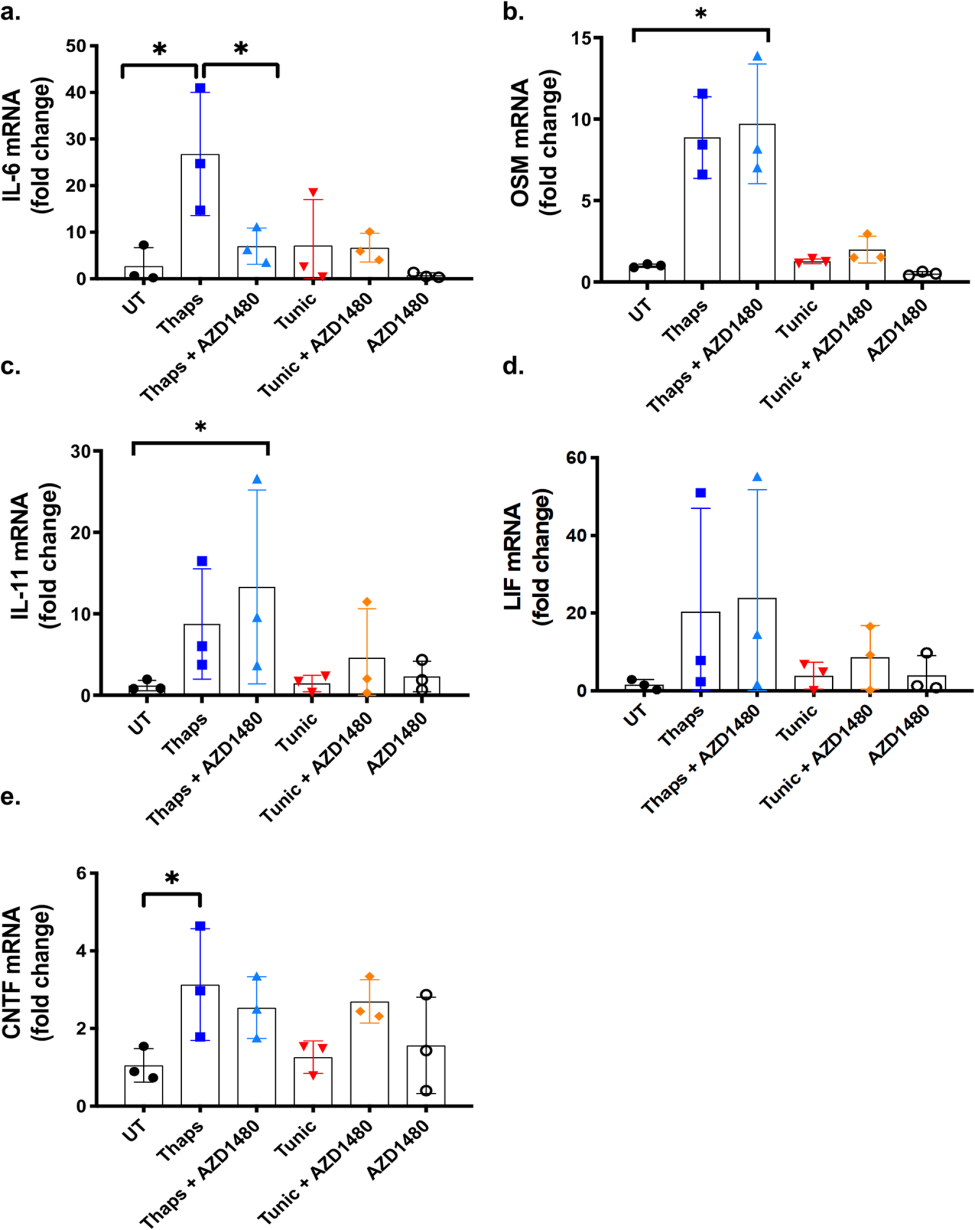
JAK-dependent regulation of the IL-6 cytokine family in macrophages. (**a**–**e**) Primary BMD murine macrophages were pre-treated for 30 min with the JAK1/2 inhibitor, AZD1480 (2 µM) followed by stimulation with 1 μM thaps and 5 μM tunic for 4 hours. mRNA expression was analyzed by qPCR. *p ≤ 0.05, N = 3.

**Figure 9:**
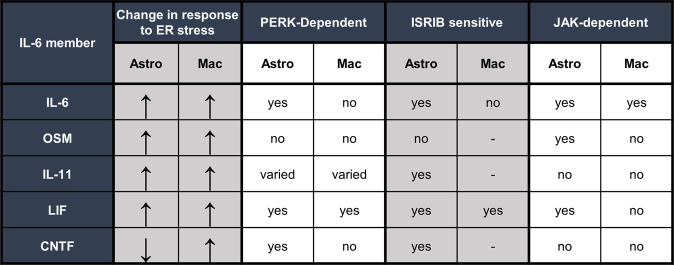
Summary of ER stress-induced regulation of the IL-6 family of cytokines in astrocytes and macrophages.

Previously, we have shown that JAK1 is an important mediator of PERK-dependent inflammatory gene expression^25^. To test if JAK signaling regulates expression of the IL-6 family of cytokines, we used the small molecule JAK1/2 inhibitor AZD1480. As shown in Fig. 7a, OSM stimulation of astrocytes robustly increases STAT3 phosphorylation at tyrosine 705, which is completely abrogated by pretreatment with AZD1480. This shows that AZD1480 effectively suppresses JAK kinase activity. Next, astrocytes were pretreated with AZD1480 followed by stimulation with thaps or tunic. AZD1480 prevented ER stress-induced IL-6 expression (Fig. 7b). AZD1480 also suppressed the expression of OSM and LIF (Fig. 7c,e) but had no effect on IL-11 or CNTF (Fig. 7d,f). These data show that JAK signaling is important for the expression of select members of the IL-6 family but is not responsible for suppression of CNTF. Interestingly, AZD1480 also potently suppressed IL-6 induction in macrophages (Fig. 8a) but did not affect other IL-6 family cytokines (Fig. 8b–e). Considering that PERK does not stimulate IL-6 expression in macrophages, this suggests that PERK may not signal to JAK1 in macrophages and that another pathway engages JAK1 to drive IL-6 expression. The mechanism by which JAK1 is activated in macrophages following ER stress is currently unknown. It is possible that JAK1 is activated secondarily to ER stress in an autocrine-dependent fashion. In this case, ER stress may quickly induce cytokines that ligate JAK1-coupled cytokine receptors leading to canonical activation of JAK/STAT signaling. STAT1 and STAT3 have been shown to synergize with NF-κB, which is an important transcription factor driving IL-6 in macrophages^39^. Thus, cytokine receptor mediated JAK1 activation leading to STAT activation may enhance IRE1-dependent IL-6 production in macrophages. Future studies will test these possibilities. Collectively, and summarized in Fig. 9, these data indicate that astrocytes and macrophages use different mechanisms to regulate the IL-6 family of cytokines in response to ER stress.

## Methods

### Animals

Wild type C57BL/6J, PERK floxed and CAGG-CreER^TM^ mice were purchased from the Jackson Laboratory and subsequently housed and bred under the care of the Office of Lab Animal Resources at West Virginia University. Mice were on a 12/12 h light/dark cycle with food and water *ad libitum*. All animal studies were conducted with approval from the WVU institutional animal care and use committee and in accordance with *Guide for the Care and Use of Laboratory Animals*.

### Astrocyte and macrophage preparation

Primary astrocytes were prepared from postnatal day 0–1 wild type C57BL/6 and PERK-CAGG-CreER^TM^ mice as previously described^40^. In brief, pups were euthanized by decapitation, and the brains were collected into cold media. Meninges, cerebellum, and olfactory bulbs were removed and cerebra were collected. Tissue was disrupted by trituration and filtered through a 100 µm cell strainer. Cells were centrifuged at 300 × g for 5 min, resuspended in fresh astrocyte medium (Dulbecco’s Modified Eagle Medium (DMEM) with 10% fetal bovine serum (FBS), 16 mM 2-[4-(2-hydroxyethyl)piperazin-1-yl]ethanesulfonic acid (HEPES), 1 × nonessential amino acids, 2 mM L-glutamine, 100 units/ml penicillin, 100 µg/ml streptomycin, and 50 µg/ml gentamicin (Fisher Scientific), and transferred onto T-75 tissue culture flasks. The cultures were maintained for approximately 12–14 days at 37 °C in humidified 5% CO_2_/95% air atmosphere. One third of the media was changed every 3–4 days. Astrocytes were separated from microglia by shaking at 200 rpm for 2 h prior to plating astrocytes in 6-well plates for experiments.

To prepare murine bone marrow-derived (BMD) macrophages, bone marrow cells were harvested from the femur and tibia of the mice, passed through a 100 µm cell strainer, and red blood cells were lysed using Ammonium-Chloride-Potassium buffer. Cells were washed with PBS and cultured for 7 days in RPMI-1640 medium (Gibco) supplemented with 10% FBS, 10% conditioned medium from L-929 cells, 2 mM glutamine, and 2.5 µg/mL penicillin/streptomycin.

### Quantitative RT-PCR analysis

Total RNA was isolated using TRIzol (Sigma Aldrich) according to manufactures instructions. RNA was quantified using a NanoDrop (ThermoFisher), and 1 μg of RNA was used for cDNA synthesis using Moloney Murine Leukemia Virus (MMLV) reverse transcriptase (Promega). The cDNA was analyzed by quantitative PCR performed using the following probe-based assays from Integrated DNA Technologies: IL-6 (193420201), IL-11 (150336742), CNTF (186082805), LIF (186082793), OSM (150336738), and Hypoxanthine Phosphoribosyltransferase 1 (HPRT) (193420197). qPCR was performed using an ABI Step One Plus (Applied Biosystems). Reactions were carried out in 20 μL, analyzed using the ΔΔCt method, and normalized to HPRT.

### Open reading frame identification

cDNA sequences were obtained from Ensemble and analyzed using NCBI ORFfinder. The IL-6 transcripts used in this analysis were human ENST00000404625.5 and mouse ENSMUST00000026845.11.

### Western blotting

Cells were washed twice in cold PBS and collected in lysis buffer (20 mM Tris [pH 7.5], 150 mM NaCl, 2 mM EDTA, 2 mM EGTA, 0.5% NP-40, and 1 × phosphatase/protease inhibitor cocktail (Pierce), and the lysates were cleared by centrifugation for 15 min at 17000 × g at 4 °C. Protein concentrations were determined using the bicinchonicic acid assay (BCA) (Pierce). Equal amounts of protein from each sample were solubilized in Laemmli sample buffer (2% SDS) and heated for 5 min at 95 °C. Proteins were separated by 8% or 10% SDS-polyacrylamide gel electrophoresis and transferred to nitrocellulose. The membranes were incubated for 1 h in 5% non-fat milk in wash buffer (20 mM Tris base, 137 mM NaCl and 0.05% Tween-20 (TBST)) at room temperature, then incubated overnight at 4 °C with the primary antibodies diluted in 5% non-fat milk or 5% BSA, per the manufacturer’s recommendation. Antibodies from Cell Signaling Technologies: anti-PERK (3192), anti-eIF2α (5324), anti-P-eIF2α (3398), anti-ATF4 (11815), anti-GADD153 (CHOP; 7351). Monoclonal mouse anti-GAPDH (MAB374) was used as a loading control (EMD Millipore). The membranes were subsequently washed and incubated with horseradish peroxidase-conjugated goat anti-mouse or anti-rabbit IgG (diluted 1:3000; Jackson Immunoresearch Laboratories) for 1 h. The immune complexes were visualized by using enhanced chemiluminescence (Thermo Scientific).

### ELISA

Cytokines were measured in 100 µl of medium according to the manufacturers’ protocol. ELISA kits for IL-6 (DY406), OSM (DY495-05), IL-11 (DY418), and LIF (DY449) were from R&D systems. ELISA for CNTF (MBS705237) was from MyBiosource.

### Statistical tests

All experiments were done at least 3 independent times. Each data point on the graphs indicates an independent experiment, boxes indicate the average and error bars are standard deviation. Analysis of the experimental data was carried out using GraphPad Prism software and one-way ANOVA followed by the Tukey’s multiple comparison test or two-way ANOVA followed by Bonferroni multiple comparison test. The level of statistical significance is defined as p < 0.05.

## Supplementary information

Endoplasmic reticulum stress differentially modulates the IL-6 family of cytokines in murine astrocytes and macrophages - supplement

## Data Availability

Data and related materials available on request.
