# Supplementary material for: Endoplasmic reticulum stress differentially modulates the IL-6 family of cytokines in murine astrocytes and macrophages
Source: Sci Rep. 2019 Oct 17;9:14931. doi: 10.1038/s41598-019-51481-6 (PMC6797742; doi:10.1038/s41598-019-51481-6)
Supplement: Supplementary file 1 — Endoplasmic reticulum stress differentially modulates the IL-6 family of cytokines in murine astrocytes and macrophages - supplement [file 41598_2019_51481_MOESM1_ESM.pdf]

Endoplasmic reticulum stress differentially modulates the IL-6 family of cytokines in murine astrocytes and macrophages.

Cristina L Sanchez[1], Savannah G Sims[1], John D Nowery[1], and Gordon P Meares[1,2*]

[1]West Virginia University, Department of Microbiology, Immunology, and Cell Biology, Morgantown, WV 26506, USA

[2]West Virginia University, Department of Neuroscience, Morgantown, WV 26506, USA

*To whom correspondence should be addressed:

Dr. Gordon Meares
West Virginia University School of Medicine
64 Medical Center Drive, HSC North 2084
Morgantown, WV 26506
304-293-6260
gpmeares@hsc.wvu.edu

Supplemental

Supplemental Figure 1

Immunoblots from figure 4

PERK

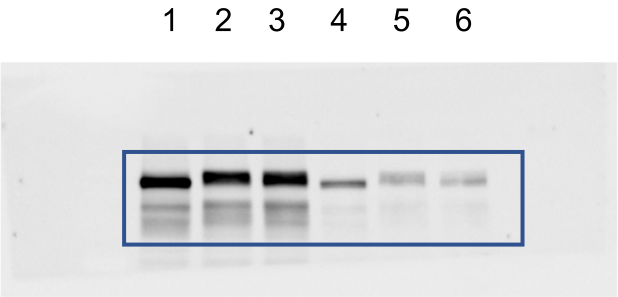

CHOP

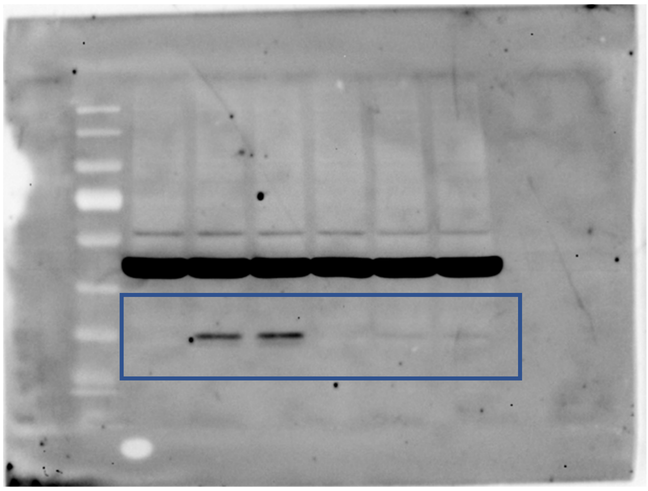

GAPDH

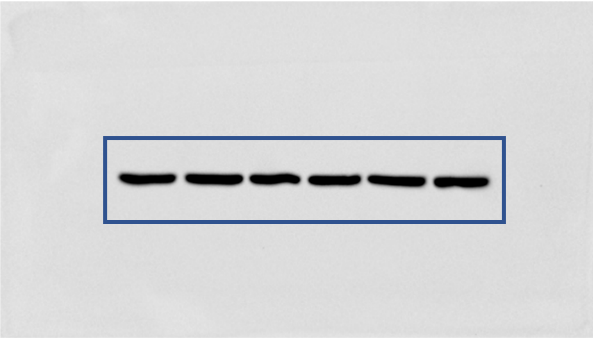

1. Cre-ER^{TM} (-) Untreated
2. Cre-ER^{TM} (-) Thapsigargin 1 μM, 4 h
3. Cre-ER^{TM} (-) Tunicamycin 5 μM, 4 h
4. Cre-ER^{TM} (+) Untreated
5. Cre-ER^{TM} (+) Thapsigargin 1 μM, 4 h
6. Cre-ER^{TM} (+) Tunicamycin 5 μM, 4 h

Supplemental Figure 2

Immunoblots from figure 5

ATF4

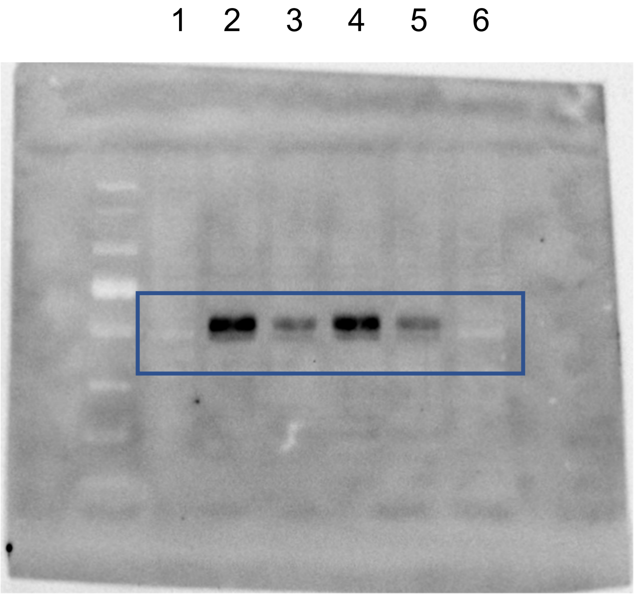

GAPDH

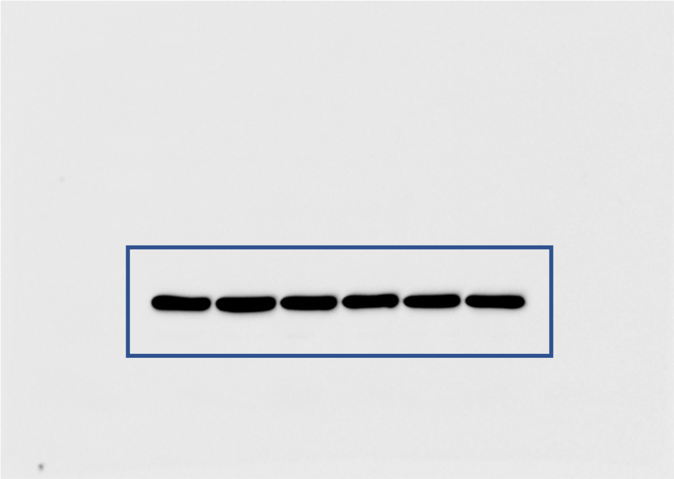

1. Untreated
2. Thapsigargin 1 μM, 4 h
3. ISRIB 0.5 μM 30 min pre + Thapsigargin 4 h
4. Tunicamycin 5 μM, 4 h
5. ISRIB 30 min pre + Tunicamycin 4 h
6. ISRIB 4.5 h

Supplemental Figure 3

Immunoblots from figure 6

PERK

1 2 3 4 5 6

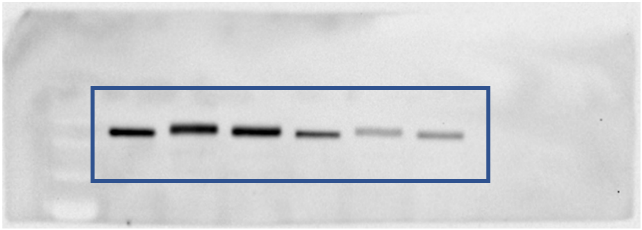

CHOP

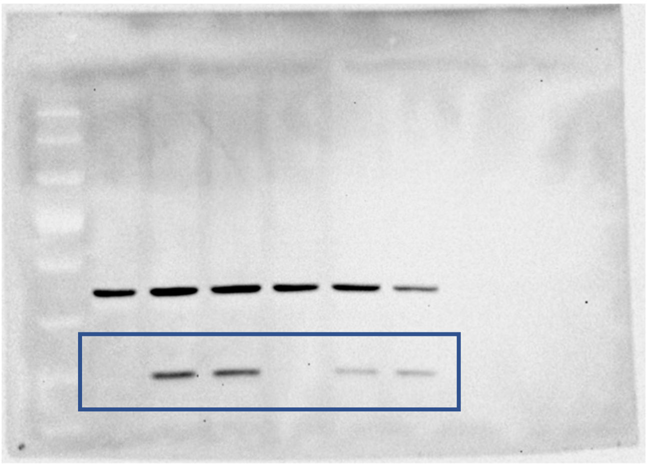

GAPDH

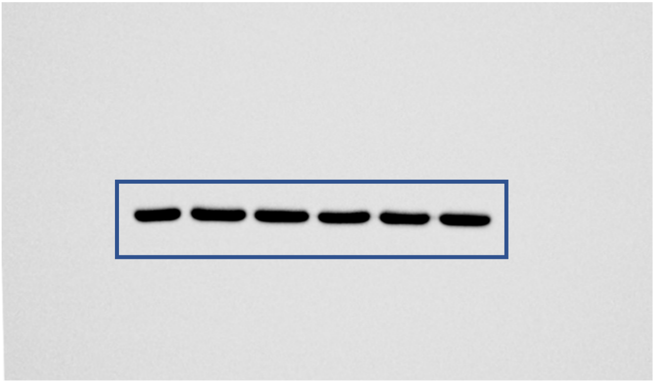

1. Cre-ER^{TM} (-) Untreated
2. Cre-ER^{TM} (-) Thapsigargin 1 μM, 4 h
3. Cre-ER^{TM} (-) Tunicamycin 5 μM, 4 h
4. Cre-ER^{TM} (+) Untreated
5. Cre-ER^{TM} (+) Thapsigargin 1 μM, 4 h
6. Cre-ER^{TM} (+) Tunicamycin 5 μM, 4 h

Supplemental Figure 4

Immunoblots from figure 7

P-STAT3

1 2 3 4

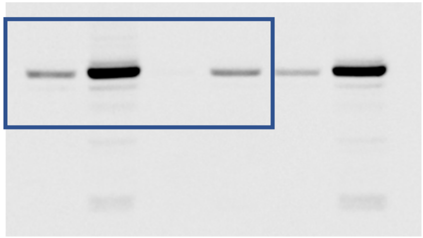

STAT3

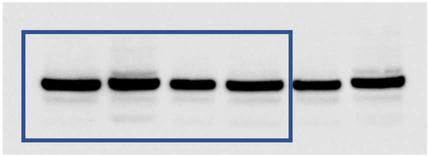

GAPDH

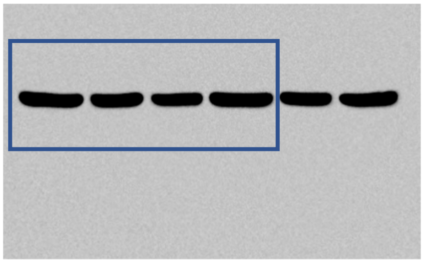

1. Untreated
2. Oncostatin M 2.5 ng/ml, 30 min
3. AZD1480 2 μM, 1 h
4. AZD1480 30 min pre + OSM 30 min
